# Cutaneous Anthrax, Belgian Traveler

**DOI:** 10.3201/eid1203.051407

**Published:** 2006-03

**Authors:** Erwin Van den Enden, Alphons Van Gompel, Marjan Van Esbroeck

**Affiliations:** * Institute of Tropical Medicine, Antwerp, Belgium

**Keywords:** Anthrax, cutaneous, Bacillus anthracis, traveler, letter

**To the Editor:** Anthrax is a rare zoonotic disease among travelers. The clinical spectrum includes cutaneous lesions, respiratory anthrax, pharyngeal inflammation, gastrointestinal infection, septicemia, and meningitis. Interest in anthrax increased after the bioterrorist attacks in the United States in 2001. The following case history describes a cutaneous infection suspected to be anthrax in a tourist who had indirect contact with dead mammals in a disease-endemic area.

After indirect contact with dead antelopes and a hippopotamus in Botswana, an acute necrotic lesion developed on a finger of a 31-year-old, healthy, female Belgian woman. The lesion became covered with a black crust, followed by massive swelling of the hand and arm. The clinical aspect and history strongly suggested cutaneous anthrax. This diagnosis was supported by seroconversion to protective antigen of *Bacillus anthracis* and the presence of antibodies against lethal factor. The bacterium itself could not be cultured or identified by polymerase chain reaction (PCR). Other members of the group with which she traveled were contacted, but no other cases were reported.

The Belgian woman traveled with friends to Namibia, Botswana, and South Africa from December 12, 2004, until January 22, 2005. She visited Chobe National Park in Botswana early January 2005. On January 8, a small, painless, vesicular lesion developed on the dorsal side of her fourth left finger. This lesion increased in size quickly and developed a black aspect with a red elevated border. Small vesicles appeared in the immediate vicinity of the primary lesion. No pus was noted. Her general condition was good. She treated herself with amoxicillin-clavulanic acid 2 gm/day for 3 days. The next day, massive edema of the finger, hand, and left arm developed. When admitted to a hospital in Johannesburg, her left arm and hand were massively swollen with painful left axillary lymphadenopathy. Her temperature never exceeded 37.8°C. Wound cultures showed only the presence of viridans streptococci, bacteria that are not implicated in wound infections. The patient was treated with intravenous ciprofloxacin, gentamicin, tetracycline, flucloxacillin, and topical mupirocin. She was discharged after 6 days with oral flucloxacillin and returned to Belgium on January 22. On February 4, her general condition was excellent; the edema had diminished. A painless necrotic lesion on the left fourth finger measured 3 cm^2^ (Figure). She mentioned minor discomfort of her left underarm and loss of sensation at the distal radial side of the left underarm. She could not extend the terminal phalanx of the fourth left finger because the underlying tendon had been destroyed. The left axillary lymph nodes were still slightly swollen. No evidence indicated parapox viral infection or necrotic arachnidism. Upon questioning, she mentioned that in Chobe National Park, some fellow travelers had manipulated the legs of dead antelopes. One person had climbed on a dead hippo for a picture and sank into the putrefying carcass. He soon afterwards cleaned a small abrasion on the patient's finger. Some hours later, all group members washed their hands in a common small plastic basin containing water and chloroxylenol.

**Figure:**
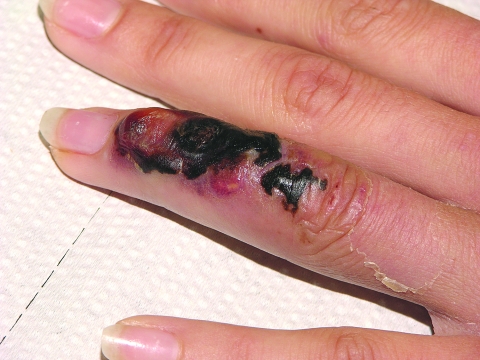
Initial skin lesion with black crust and red border, suggestive of cutaneous anthrax. By the time the picture was taken, the massive edema of hand and arm had subsided.

Full blood count, erythrocyte sedimentation rate, and biochemistry were normal. Antistreptolysin O levels were within normal limits. Serologic test results for rickettsiae, orthopoxviruses, and *Bartonella henselae* were negative. The patient was not immunocompromised. Because cutaneous anthrax was suspected, wound crusts, swabs for bacterial cultures, and Dacron swabs used for PCR were mailed as quickly as possible to the Belgian national reference laboratory. All cultures remained sterile. PCR was negative for *B. anthracis*. Because of the positive clinical outcome with antimicrobial drugs for 16 days, no additional antimicrobial drugs or steroids were prescribed. Further recovery was uneventful and only a small scar remains. While waiting for serologic test results, a ProMed alert was issued (*1*). Members of the travel group were contacted and warned but no other cases were identified. Consecutive serum samples were analyzed for *B. anthracis* protective antigen antibodies (anti-PA) (Centers for Disease Control and Prevention, Atlanta, GA, USA). The serum collected on February 4 was negative. On February 16, anti-PA immunoglobulin G (IgG) was detected with a titer of 9.5 (weakly positive). On April 18, no anti-PA IgG could be detected. Paired serum samples (February 4 and 16) were also mailed to the Institut für Microbiologie der Bundeswehr in Munich, Germany. In the German laboratory, the anti-PA enzyme-linked immunosorbent assay result was negative, but specific antibodies against lethal factor of *B. anthracis* were detected.

Anthrax is essentially a disease of grazing animals and is relatively common in persons who have contact with these animals (*2**–**4*). It is occasionally reported in travelers (*5*). In this case, many arguments existed for cutaneous anthrax, but the diagnosis could not be proven. Clinical symptoms (malignant edema) and history of indirect contact with carcasses of wildlife in a disease-endemic area suggested anthrax. Bacterial cultures remained negative, presumably because of previous administration of antimicrobial drugs. The clinical diagnosis was supported by seroconversion to protective antigen and the presence of antibodies against lethal factor. In cutaneous anthrax, antibodies to protective antigen develop in 68%–92% of cases (*6**,**7*). Previous cases of cutaneous anthrax in Belgium date from the 1980s, when a man became infected while unloading Indian bone meal in Antwerp Harbor. In 1986, cutaneous anthrax developed in a Turkish woman after being injured while cooking a sheep (*8*). In 2002, a suspected case in a Belgian farmer was reported (*9*). Many cases of cutaneous anthrax heal spontaneously, but a 5%–10% chance of systemic complications exists. This case illustrates 1 of the dangers of touching dead animals in nature. Travelers should be warned that even indirect contact can lead to problems.
